# Whole-mol­ecule disordered (*E*)-2-(1-hy­droxy-3-phenylprop-2-en-1-yl­idene)-4,5-dimeth­oxycyclo­pent-4-ene-1,3-dione isolated from *Lindera oxyphylla* (Lauraceae)

**DOI:** 10.1107/S1600536811019386

**Published:** 2011-05-28

**Authors:** Masoumeh Hosseinzadeh, Mat Ropi Mukhtar, Jamaludin Mohamad, Khalijah Awang, Seik Weng Ng

**Affiliations:** a Department of Chemistry, University of Malaya, 50603 Kuala Lumpur, Malaysia; b Institute of Biological Sciences, University of Malaya, 50603 Kuala Lumpur, Malaysia

## Abstract

In the mol­ecule of the title compound, C_16_H_14_O_5_, all non-H atoms are approximately co-planar [maximum atomic deviation = 0.064 (5) Å]. The hy­droxy group is a hydrogen-bond donor to a carbonyl O atom. Weak intermolecular C—H⋯O hydrogen bonding is present in the crystal structure. The crystal structure is ’whole-mol­ecule disordered’ about an axis that runs approximately along the length of the mol­ecule; the occupancy of the two disorder components was set as exactly 0.5. An intra­molecular O—-H⋯O hydrogen bond exists in each component.

## Related literature

For the spectroscopic characterization of linderone and methyl linderone isolated from *Lindera pipericarpa*, see: Kiang *et al.* (1962 ▶). For the crystal structure of methyl linderone isolated from *Lindera poliantha*, see: Syah *et al.* (2005 ▶).
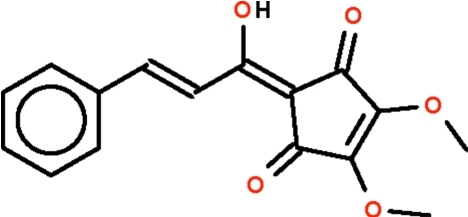


## Experimental

### 

#### Crystal data

C_16_H_14_O_5_
*M* *_r_* = 286.27
Monoclinic, 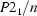

*a* = 7.3195 (5) Å
*b* = 9.8635 (7) Å
*c* = 18.6724 (11) Å
β = 96.586 (6)°
*V* = 1339.17 (15) Å^3^
*Z* = 4
Mo *K*α radiation
μ = 0.11 mm^−1^
*T* = 100 K
0.20 × 0.20 × 0.10 mm

#### Data collection

Agilent SuperNova Dual diffractometer with an Atlas detector
Absorption correction: multi-scan (*CrysAlis PRO*; Agilent, 2010 ▶) *T*_min_ = 0.979, *T*_max_ = 0.990
8436 measured reflections
2369 independent reflections
1965 reflections with *I* > 2σ(*I*)
*R*_int_ = 0.032

#### Refinement

*R*[*F* ^2^ > 2σ(*F* ^2^)] = 0.068
*wR*(*F* ^2^) = 0.190
*S* = 1.05
2369 reflections
308 parameters
30 restraints
H-atom parameters constrained
Δρ_max_ = 0.56 e Å^−3^
Δρ_min_ = −0.34 e Å^−3^

### 

Data collection: *CrysAlis PRO* (Agilent, 2010 ▶); cell refinement: *CrysAlis PRO*; data reduction: *CrysAlis PRO*; program(s) used to solve structure: *SHELXS97* (Sheldrick, 2008 ▶); program(s) used to refine structure: *SHELXL97* (Sheldrick, 2008 ▶); molecular graphics: *X-SEED* (Barbour, 2001 ▶); software used to prepare material for publication: *publCIF* (Westrip, 2010 ▶).

## Supplementary Material

Crystal structure: contains datablocks global, I. DOI: 10.1107/S1600536811019386/xu5217sup1.cif

Structure factors: contains datablocks I. DOI: 10.1107/S1600536811019386/xu5217Isup2.hkl

Supplementary material file. DOI: 10.1107/S1600536811019386/xu5217Isup3.cml

Additional supplementary materials:  crystallographic information; 3D view; checkCIF report

## Figures and Tables

**Table 1:** Hydrogen-bond geometry (Å, °)

*D*—H⋯*A*	*D*—H	H⋯*A*	*D*⋯*A*	*D*—H⋯*A*
O1—H1⋯O2	0.84	1.93	2.600 (5)	136
O1′—H1′⋯O2′	0.84	1.97	2.623 (4)	134
C2—H2⋯O2^i^	0.95	2.26	3.091 (8)	145
C16′—H16*D*⋯O1′^ii^	0.98	2.05	2.885 (11)	142

Symmetry codes: (i) 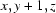; (ii) 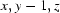.

**Fig. 1.**
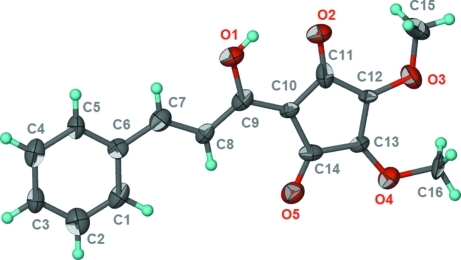
Thermal ellipsoid plot (Barbour, 2001) of one of the whole-molcule disordered components of C16H14O5 at the 70% probability level; hydrogen atoms are drawn as spheres of arbitrary radius.

## supplementary crystallographic information

### Comment

Linderone (Scheme I) was isolated from *Lindera pipericarpa* and its formulation was established by solution 1*H*-NMR spectrocopy (Kiang *et al.*, 1962) nearly 50 years ago. This plant genus also yields methyl linderone, which differs from linderone in having a methyl group in place of the hydroxy H atom. Methyl linderone, isolated from *Lindera poliantha*, exists as a planar molecule (Syah *et al.*, 2005). Linderone is similarly a planar molecule; however, the molecule is 'whole-molecule' disordered (Fig. 1) about an axis that runs approximately along the length of the flat molecule. The three-atom chain connecting the five-membered and six-membered rings exists in an *E*-configuration; the hydroxy group is hydrogen-bond donor to the carbonyl O atom.

### Experimental

*Lindera oxyphylla* (Lauraceae) was collected from Ulu Muda, Baling, Kedah, Malaysia. Some 4 kg of dried and ground bark of *Lindera oxyphylla* were extracted with hexane (10 *L*) for 3 days. The hexane extract was concentrated under reduced pressure to give a crude material (13 g). This was subjected to column chromatography on silica gel-60 (2 *x* 75 cm, 70–230 mesh ASTM) by using a step gradient of hexane and dichloromethane. The separation afforded 30 fractions; fractions 22–30 were purified by using dichloromethane–methanol (98:2) to yield (*E*)- 2-(1-hydroxy-3-phenyl-2-propen-1-ylidene)-4,5-dimethoxy-4-cyclopentene-1,3-dione. Its formulation was established by solution NMR spectroscopic analysis. Deep yellow prisms were obtained upon recrystallization from dichloromethane.

### Refinement

Carbon- and oxygen-bound H-atoms were placed in calculated positions [C—H 0.95 to 0.98. O–H 0.84 Å, *U*_iso_(H) 1.2 to 1.5*U*_eq_(C,O)], and were included in the refinement in the riding model approximation. An *sp*^2^-type of hybridization was assumed for the hydroxy H atom.

The crystal structure is a 'whole-molecule disordered' crystal structure. As the occupancy refined to near 1:1, the occupancy of the two disorder components was set as exactly 0.5.

The phenyl ring was refined as a rigid hexagon of 1.39 Å sides and the five-membered ring a rigid pentagon of 1.42 Å sides. The temperature factors of the atoms constituting the five-membered ring were set to those of the umprimed ones, and the anisotropic temperature factors were restrained to be nearly isotropic.

The extinction was refined; although the value is small, its refinement improved the refinement somewhat.

The crystal used for the measurements was a twinned crystal of low mosaicity; fortunately, the presence of the minor twin component did not affect the diffraction intensities of the major component only the diffraction intensities of the major component were integrated. On the other hand, the simultaneous integration of both components lead to a less satisfactory refinement. Other crystals were also measured but these demonstrated varying mosaicities and degrees of twinning (from 0 to 50%), and neither were the refinements improved by the use of copper radiation in place of molybdenum radiation.

### Crystal data

**Table:**

C_16_H_14_O_5_	*F*(000) = 600
*M**_r_* = 286.27	*D*_x_ = 1.420 Mg m^−^^3^
Monoclinic, *P*2_1_/*n*	Mo *K*α radiation, λ = 0.71073 Å
Hall symbol: -P 2yn	Cell parameters from 4706 reflections
*a* = 7.3195 (5) Å	θ = 2.2–25.0°
*b* = 9.8635 (7) Å	µ = 0.11 mm^−^^1^
*c* = 18.6724 (11) Å	*T* = 100 K
β = 96.586 (6)°	Prism, yellow
*V* = 1339.17 (15) Å^3^	0.20 × 0.20 × 0.10 mm
*Z* = 4	

### Data collection

**Table:**

Agilent SuperNova Dual diffractometer with an Atlas detector	2369 independent reflections
Radiation source: SuperNova (Mo) X-ray Source	1965 reflections with *I* > 2σ(*I*)
Mirror	*R*_int_ = 0.032
Detector resolution: 10.4041 pixels mm^-1^	θ_max_ = 25.1°, θ_min_ = 2.2°
ω scans	*h* = −8→8
Absorption correction: multi-scan (*CrysAlis PRO*; Agilent, 2010)	*k* = −11→10
*T*_min_ = 0.979, *T*_max_ = 0.990	*l* = −22→22
8436 measured reflections	

### Refinement

**Table:**

Refinement on *F*^2^	Secondary atom site location: difference Fourier map
Least-squares matrix: full	Hydrogen site location: inferred from neighbouring sites
*R*[*F*^2^ > 2σ(*F*^2^)] = 0.068	H-atom parameters constrained
*wR*(*F*^2^) = 0.190	*w* = 1/[σ^2^(*F*_o_^2^) + (0.0883*P*)^2^ + 1.2962*P*] where *P* = (*F*_o_^2^ + 2*F*_c_^2^)/3
*S* = 1.05	(Δ/σ)_max_ = 0.001
2369 reflections	Δρ_max_ = 0.56 e Å^−^^3^
308 parameters	Δρ_min_ = −0.34 e Å^−^^3^
30 restraints	Extinction correction: *SHELXL97* (Sheldrick, 2008), Fc^*^=kFc[1+0.001xFc^2^λ^3^/sin(2θ)]^-1/4^
Primary atom site location: structure-invariant direct methods	Extinction coefficient: 0.0014 (11)

### Fractional atomic coordinates and isotropic or equivalent isotropic displacement parameters (Å^2^)

**Table:**

	*x*	*y*	*z*	*U*_iso_*/*U*_eq_	Occ. (<1)
O1	0.1902 (5)	0.2729 (4)	0.58692 (19)	0.0387 (8)	0.50
H1	0.1994	0.1894	0.5793	0.058*	0.50
O2	0.2649 (5)	0.0774 (4)	0.50156 (19)	0.0417 (9)	0.50
O3	0.3802 (5)	0.0354 (4)	0.3530 (2)	0.0405 (9)	0.50
O4	0.4003 (4)	0.3167 (3)	0.29148 (16)	0.0326 (8)	0.50
O5	0.3180 (4)	0.5067 (4)	0.39499 (17)	0.0341 (8)	0.50
C1	0.1608 (5)	0.7931 (7)	0.6029 (2)	0.0322 (14)	0.50
H1a	0.1943	0.7828	0.5555	0.039*	0.50
C2	0.1349 (12)	0.9219 (5)	0.6302 (4)	0.038 (2)	0.50
H2	0.1507	0.9996	0.6015	0.046*	0.50
C3	0.0859 (18)	0.9370 (5)	0.6995 (5)	0.031 (3)	0.50
H3	0.0682	1.0250	0.7182	0.037*	0.50
C4	0.0629 (17)	0.8233 (7)	0.7415 (3)	0.033 (3)	0.50
H4	0.0294	0.8336	0.7889	0.040*	0.50
C5	0.0888 (9)	0.6945 (6)	0.7142 (3)	0.0282 (16)	0.50
H5	0.0730	0.6168	0.7429	0.034*	0.50
C6	0.1377 (5)	0.6794 (5)	0.6449 (3)	0.0245 (16)	0.50
C7	0.1602 (6)	0.5419 (5)	0.6182 (2)	0.0302 (10)	0.50
H7	0.1380	0.4705	0.6503	0.036*	0.50
C8	0.2094 (11)	0.5036 (8)	0.5527 (4)	0.0241 (15)	0.50
H8	0.2331	0.5701	0.5182	0.029*	0.50
C9	0.2249 (6)	0.3619 (5)	0.5364 (2)	0.0284 (10)	0.50
C10	0.2768 (5)	0.3111 (4)	0.47099 (16)	0.0217 (10)	0.50
C11	0.2933 (6)	0.1698 (3)	0.45873 (17)	0.0324 (11)	0.50
C12	0.3440 (6)	0.1517 (3)	0.38819 (19)	0.0183 (11)	0.50
C13	0.3588 (5)	0.2819 (4)	0.35685 (14)	0.0219 (9)	0.50
C14	0.3172 (5)	0.3804 (2)	0.4080 (2)	0.0254 (8)	0.50
C15	0.3509 (17)	−0.0907 (11)	0.3878 (5)	0.051 (2)	0.50
H15A	0.3823	−0.1658	0.3571	0.077*	0.50
H15B	0.2215	−0.0980	0.3962	0.077*	0.50
H15C	0.4289	−0.0947	0.4341	0.077*	0.50
C16	0.4447 (17)	0.2108 (10)	0.2420 (6)	0.032 (2)	0.50
H16A	0.4725	0.2520	0.1968	0.049*	0.50
H16B	0.3396	0.1494	0.2323	0.049*	0.50
H16C	0.5519	0.1597	0.2637	0.049*	0.50
O1'	0.3120 (4)	0.6031 (3)	0.42276 (16)	0.0284 (7)	0.50
H1'	0.3434	0.5848	0.3820	0.043*	0.50
O2'	0.3866 (4)	0.4149 (3)	0.33121 (15)	0.0286 (7)	0.50
O3'	0.3939 (4)	0.1087 (4)	0.31727 (17)	0.0303 (7)	0.50
O4'	0.2841 (5)	0.0061 (3)	0.46004 (18)	0.0356 (8)	0.50
O5'	0.2174 (5)	0.2272 (3)	0.54512 (19)	0.0349 (8)	0.50
C1'	0.1246 (5)	0.6426 (4)	0.6802 (3)	0.0236 (11)	0.50
H1'a	0.1350	0.5471	0.6754	0.028*	0.50
C2'	0.0771 (10)	0.6978 (7)	0.7440 (3)	0.0290 (16)	0.50
H2'	0.0550	0.6401	0.7828	0.035*	0.50
C3'	0.0618 (17)	0.8376 (7)	0.7510 (4)	0.031 (2)	0.50
H3'	0.0293	0.8753	0.7946	0.037*	0.50
C4'	0.0942 (18)	0.9221 (4)	0.6942 (5)	0.036 (3)	0.50
H4'	0.0838	1.0175	0.6990	0.043*	0.50
C5'	0.1418 (11)	0.8668 (5)	0.6304 (3)	0.0274 (16)	0.50
H5'	0.1639	0.9246	0.5916	0.033*	0.50
C6'	0.1570 (5)	0.7271 (5)	0.6234 (2)	0.0202 (15)	0.50
C7'	0.2082 (5)	0.6752 (4)	0.5545 (2)	0.0220 (9)	0.50
H7'	0.2306	0.7410	0.5193	0.026*	0.50
C8'	0.2262 (12)	0.5464 (7)	0.5363 (5)	0.0237 (15)	0.50
H8'	0.2028	0.4789	0.5704	0.028*	0.50
C9'	0.2790 (5)	0.5022 (4)	0.4682 (2)	0.0219 (9)	0.50
C10'	0.2968 (5)	0.3663 (3)	0.4479 (2)	0.0217 (10)	0.50
C11'	0.3503 (5)	0.3338 (3)	0.37918 (18)	0.0324 (11)	0.50
C12'	0.3537 (6)	0.1904 (3)	0.37286 (15)	0.0183 (11)	0.50
C13'	0.3024 (6)	0.1342 (2)	0.43766 (18)	0.0219 (9)	0.50
C14'	0.2673 (5)	0.2429 (4)	0.48403 (13)	0.0254 (8)	0.50
C15'	0.4107 (15)	0.1709 (10)	0.2489 (7)	0.034 (2)	0.50
H15D	0.4407	0.1017	0.2145	0.051*	0.50
H15E	0.5088	0.2391	0.2546	0.051*	0.50
H15F	0.2942	0.2146	0.2309	0.051*	0.50
C16'	0.3136 (18)	−0.1051 (11)	0.4120 (5)	0.054 (3)	0.50
H16D	0.2954	−0.1914	0.4362	0.081*	0.50
H16E	0.4394	−0.1009	0.3989	0.081*	0.50
H16F	0.2260	−0.0983	0.3683	0.081*	0.50

### Atomic displacement parameters (Å^2^)

**Table:**

	*U*^11^	*U*^22^	*U*^33^	*U*^12^	*U*^13^	*U*^23^
O1	0.053 (2)	0.035 (2)	0.0288 (19)	0.0007 (16)	0.0099 (16)	0.0090 (16)
O2	0.061 (2)	0.034 (2)	0.0326 (18)	0.0009 (17)	0.0146 (16)	0.0108 (17)
O3	0.056 (2)	0.0259 (19)	0.042 (2)	0.0032 (16)	0.0153 (17)	−0.0052 (17)
O4	0.0370 (18)	0.0352 (19)	0.0273 (16)	0.0001 (14)	0.0109 (13)	0.0018 (15)
O5	0.0366 (18)	0.037 (2)	0.0295 (17)	0.0010 (15)	0.0076 (14)	0.0037 (16)
C1	0.025 (3)	0.051 (5)	0.021 (3)	−0.001 (3)	0.005 (2)	−0.001 (3)
C2	0.031 (3)	0.041 (5)	0.043 (4)	−0.003 (4)	0.006 (3)	−0.006 (4)
C3	0.030 (5)	0.037 (4)	0.026 (5)	0.002 (4)	0.006 (4)	−0.009 (4)
C4	0.021 (5)	0.049 (7)	0.029 (3)	−0.005 (4)	0.004 (3)	−0.010 (4)
C5	0.028 (3)	0.032 (3)	0.025 (4)	0.000 (2)	0.006 (3)	−0.008 (4)
C6	0.020 (2)	0.028 (4)	0.027 (4)	−0.004 (2)	0.007 (2)	0.002 (3)
C7	0.026 (2)	0.038 (3)	0.026 (2)	−0.0021 (19)	0.0014 (17)	0.000 (2)
C8	0.023 (3)	0.032 (4)	0.018 (3)	0.001 (3)	0.004 (2)	0.001 (3)
C9	0.025 (2)	0.039 (3)	0.022 (2)	0.0005 (19)	0.0043 (17)	0.0036 (19)
C10	0.0244 (15)	0.022 (2)	0.019 (2)	0.0003 (17)	0.0042 (14)	0.0059 (18)
C11	0.034 (2)	0.033 (2)	0.030 (2)	0.0013 (18)	0.0023 (17)	−0.0119 (19)
C12	0.0202 (12)	0.015 (2)	0.0199 (18)	−0.0023 (15)	0.0056 (12)	0.0068 (19)
C13	0.0227 (16)	0.0248 (19)	0.0181 (17)	−0.0019 (14)	0.0022 (14)	0.0051 (16)
C14	0.0220 (16)	0.0245 (19)	0.029 (2)	−0.0042 (14)	−0.0012 (14)	0.0090 (17)
C15	0.077 (5)	0.023 (4)	0.057 (7)	0.008 (3)	0.024 (5)	0.006 (4)
C16	0.049 (5)	0.034 (5)	0.017 (3)	−0.011 (3)	0.011 (3)	−0.012 (3)
O1'	0.0424 (18)	0.0215 (17)	0.0233 (15)	−0.0008 (13)	0.0116 (13)	−0.0016 (13)
O2'	0.0358 (17)	0.0301 (18)	0.0210 (15)	−0.0019 (13)	0.0086 (12)	−0.0009 (13)
O3'	0.0394 (18)	0.0311 (18)	0.0220 (16)	0.0042 (14)	0.0098 (13)	−0.0035 (15)
O4'	0.055 (2)	0.0233 (17)	0.0293 (17)	0.0025 (15)	0.0097 (15)	−0.0069 (15)
O5'	0.058 (2)	0.0294 (19)	0.0205 (17)	−0.0003 (15)	0.0178 (16)	−0.0020 (15)
C1'	0.029 (2)	0.021 (3)	0.023 (3)	−0.003 (2)	0.011 (2)	0.003 (3)
C2'	0.030 (3)	0.035 (4)	0.024 (4)	−0.001 (2)	0.009 (3)	−0.009 (3)
C3'	0.028 (5)	0.028 (5)	0.036 (4)	−0.002 (3)	0.002 (3)	−0.012 (4)
C4'	0.026 (5)	0.026 (4)	0.055 (8)	−0.001 (3)	0.002 (5)	−0.013 (4)
C5'	0.028 (3)	0.029 (5)	0.025 (3)	0.000 (3)	0.005 (2)	−0.004 (3)
C6'	0.020 (2)	0.023 (4)	0.018 (3)	−0.002 (2)	0.003 (2)	0.006 (3)
C7'	0.020 (2)	0.025 (2)	0.021 (2)	−0.0014 (16)	0.0053 (16)	0.0032 (17)
C8'	0.027 (3)	0.018 (4)	0.026 (4)	0.001 (3)	0.003 (2)	0.007 (2)
C9'	0.0173 (19)	0.025 (2)	0.023 (2)	−0.0011 (16)	0.0002 (16)	0.0006 (17)
C10'	0.0244 (15)	0.022 (2)	0.019 (2)	0.0003 (17)	0.0042 (14)	0.0059 (18)
C11'	0.034 (2)	0.033 (2)	0.030 (2)	0.0013 (18)	0.0023 (17)	−0.0119 (19)
C12'	0.0202 (12)	0.015 (2)	0.0199 (18)	−0.0023 (15)	0.0056 (12)	0.0068 (19)
C13'	0.0227 (16)	0.0248 (19)	0.0181 (17)	−0.0019 (14)	0.0022 (14)	0.0051 (16)
C14'	0.0220 (16)	0.0245 (19)	0.029 (2)	−0.0042 (14)	−0.0012 (14)	0.0090 (17)
C15'	0.025 (4)	0.042 (6)	0.035 (4)	0.000 (4)	0.011 (3)	−0.010 (4)
C16'	0.101 (8)	0.021 (4)	0.041 (6)	0.001 (4)	0.017 (4)	−0.011 (4)

### Geometric parameters (Å, °)

**Table:**

O1—C9	1.334 (6)	O1'—C9'	1.347 (5)
O1—H1	0.8400	O1'—H1'	0.8400
O2—C11	1.246 (4)	O2'—C11'	1.252 (4)
O3—C12	1.364 (4)	O3'—C12'	1.373 (4)
O3—C15	1.431 (10)	O3'—C15'	1.434 (11)
O4—C13	1.336 (4)	O4'—C13'	1.342 (4)
O4—C16	1.455 (11)	O4'—C16'	1.449 (10)
O5—C14	1.269 (4)	O5'—C14'	1.247 (4)
C1—C2	1.3900	C1'—C2'	1.3900
C1—C6	1.3900	C1'—C6'	1.3900
C1—H1A	0.9500	C1'—H1'A	0.9500
C2—C3	1.3900	C2'—C3'	1.3900
C2—H2	0.9500	C2'—H2'	0.9500
C3—C4	1.3900	C3'—C4'	1.3900
C3—H3	0.9500	C3'—H3'	0.9500
C4—C5	1.3900	C4'—C5'	1.3900
C4—H4	0.9500	C4'—H4'	0.9500
C5—C6	1.3900	C5'—C6'	1.3900
C5—H5	0.9500	C5'—H5'	0.9500
C6—C7	1.460 (6)	C6'—C7'	1.473 (5)
C7—C8	1.367 (10)	C7'—C8'	1.326 (10)
C7—H7	0.9500	C7'—H7'	0.9500
C8—C9	1.437 (10)	C8'—C9'	1.438 (10)
C8—H8	0.9500	C8'—H8'	0.9500
C9—C10	1.412 (5)	C9'—C10'	1.404 (5)
C10—C11	1.4200	C10'—C11'	1.4200
C10—C14	1.4200	C10'—C14'	1.4200
C11—C12	1.4200	C11'—C12'	1.4200
C12—C13	1.4200	C12'—C13'	1.4200
C13—C14	1.4200	C13'—C14'	1.4200
C15—H15A	0.9800	C15'—H15D	0.9800
C15—H15B	0.9800	C15'—H15E	0.9800
C15—H15C	0.9800	C15'—H15F	0.9800
C16—H16A	0.9800	C16'—H16D	0.9800
C16—H16B	0.9800	C16'—H16E	0.9800
C16—H16C	0.9800	C16'—H16F	0.9800
			
C9—O1—H1	120.0	C9'—O1'—H1'	120.0
C12—O3—C15	117.7 (5)	C12'—O3'—C15'	118.0 (5)
C13—O4—C16	119.1 (5)	C13'—O4'—C16'	119.5 (5)
C2—C1—C6	120.0	C2'—C1'—C6'	120.0
C2—C1—H1A	120.0	C2'—C1'—H1'A	120.0
C6—C1—H1A	120.0	C6'—C1'—H1'A	120.0
C1—C2—C3	120.0	C3'—C2'—C1'	120.0
C1—C2—H2	120.0	C3'—C2'—H2'	120.0
C3—C2—H2	120.0	C1'—C2'—H2'	120.0
C4—C3—C2	120.0	C2'—C3'—C4'	120.0
C4—C3—H3	120.0	C2'—C3'—H3'	120.0
C2—C3—H3	120.0	C4'—C3'—H3'	120.0
C5—C4—C3	120.0	C5'—C4'—C3'	120.0
C5—C4—H4	120.0	C5'—C4'—H4'	120.0
C3—C4—H4	120.0	C3'—C4'—H4'	120.0
C4—C5—C6	120.0	C4'—C5'—C6'	120.0
C4—C5—H5	120.0	C4'—C5'—H5'	120.0
C6—C5—H5	120.0	C6'—C5'—H5'	120.0
C5—C6—C1	120.0	C5'—C6'—C1'	120.0
C5—C6—C7	117.9 (5)	C5'—C6'—C7'	117.3 (4)
C1—C6—C7	122.1 (5)	C1'—C6'—C7'	122.7 (4)
C8—C7—C6	127.8 (5)	C8'—C7'—C6'	126.8 (5)
C8—C7—H7	116.1	C8'—C7'—H7'	116.6
C6—C7—H7	116.1	C6'—C7'—H7'	116.6
C7—C8—C9	119.6 (6)	C7'—C8'—C9'	124.1 (5)
C7—C8—H8	120.2	C7'—C8'—H8'	117.9
C9—C8—H8	120.2	C9'—C8'—H8'	117.9
O1—C9—C10	118.1 (4)	O1'—C9'—C10'	120.4 (4)
O1—C9—C8	117.6 (5)	O1'—C9'—C8'	114.8 (4)
C10—C9—C8	124.3 (5)	C10'—C9'—C8'	124.8 (4)
C9—C10—C11	121.6 (4)	C9'—C10'—C11'	120.2 (3)
C9—C10—C14	130.4 (4)	C9'—C10'—C14'	131.8 (3)
C11—C10—C14	108.0	C11'—C10'—C14'	108.0
O2—C11—C12	125.7 (4)	O2'—C11'—C12'	124.7 (3)
O2—C11—C10	126.2 (4)	O2'—C11'—C10'	127.3 (3)
C12—C11—C10	108.0	C12'—C11'—C10'	108.0
O3—C12—C13	122.3 (3)	O3'—C12'—C11'	131.0 (3)
O3—C12—C11	129.7 (3)	O3'—C12'—C13'	121.0 (3)
C13—C12—C11	108.0	C11'—C12'—C13'	108.0
O4—C13—C12	130.1 (3)	O4'—C13'—C14'	119.3 (3)
O4—C13—C14	121.9 (3)	O4'—C13'—C12'	132.7 (3)
C12—C13—C14	108.0	C14'—C13'—C12'	108.0
O5—C14—C13	122.4 (4)	O5'—C14'—C13'	123.8 (3)
O5—C14—C10	129.6 (4)	O5'—C14'—C10'	128.2 (3)
C13—C14—C10	108.0	C13'—C14'—C10'	108.0
O3—C15—H15A	109.5	O3'—C15'—H15D	109.5
O3—C15—H15B	109.5	O3'—C15'—H15E	109.5
H15A—C15—H15B	109.5	H15D—C15'—H15E	109.5
O3—C15—H15C	109.5	O3'—C15'—H15F	109.5
H15A—C15—H15C	109.5	H15D—C15'—H15F	109.5
H15B—C15—H15C	109.5	H15E—C15'—H15F	109.5
O4—C16—H16A	109.5	O4'—C16'—H16D	109.5
O4—C16—H16B	109.5	O4'—C16'—H16E	109.5
H16A—C16—H16B	109.5	H16D—C16'—H16E	109.5
O4—C16—H16C	109.5	O4'—C16'—H16F	109.5
H16A—C16—H16C	109.5	H16D—C16'—H16F	109.5
H16B—C16—H16C	109.5	H16E—C16'—H16F	109.5
			
C6—C1—C2—C3	0.0	C6'—C1'—C2'—C3'	0.0
C1—C2—C3—C4	0.0	C1'—C2'—C3'—C4'	0.0
C2—C3—C4—C5	0.0	C2'—C3'—C4'—C5'	0.0
C3—C4—C5—C6	0.0	C3'—C4'—C5'—C6'	0.0
C4—C5—C6—C1	0.0	C4'—C5'—C6'—C1'	0.0
C4—C5—C6—C7	−179.0 (5)	C4'—C5'—C6'—C7'	−179.9 (5)
C2—C1—C6—C5	0.0	C2'—C1'—C6'—C5'	0.0
C2—C1—C6—C7	178.9 (5)	C2'—C1'—C6'—C7'	179.9 (5)
C5—C6—C7—C8	−179.2 (6)	C5'—C6'—C7'—C8'	−178.5 (6)
C1—C6—C7—C8	1.9 (7)	C1'—C6'—C7'—C8'	1.6 (7)
C6—C7—C8—C9	179.8 (5)	C6'—C7'—C8'—C9'	−179.2 (5)
C7—C8—C9—O1	−0.1 (9)	C7'—C8'—C9'—O1'	0.7 (9)
C7—C8—C9—C10	−179.2 (5)	C7'—C8'—C9'—C10'	−179.3 (6)
O1—C9—C10—C11	−0.4 (5)	O1'—C9'—C10'—C11'	0.1 (5)
C8—C9—C10—C11	178.7 (5)	C8'—C9'—C10'—C11'	−179.9 (5)
O1—C9—C10—C14	178.4 (3)	O1'—C9'—C10'—C14'	−178.5 (3)
C8—C9—C10—C14	−2.5 (7)	C8'—C9'—C10'—C14'	1.5 (7)
C9—C10—C11—O2	0.4 (5)	C9'—C10'—C11'—O2'	0.2 (5)
C14—C10—C11—O2	−178.6 (5)	C14'—C10'—C11'—O2'	179.2 (4)
C9—C10—C11—C12	179.0 (4)	C9'—C10'—C11'—C12'	−178.9 (4)
C14—C10—C11—C12	0.0	C14'—C10'—C11'—C12'	0.0
C15—O3—C12—C13	−176.5 (6)	C15'—O3'—C12'—C11'	−10.5 (7)
C15—O3—C12—C11	5.5 (8)	C15'—O3'—C12'—C13'	168.5 (5)
O2—C11—C12—O3	−3.2 (5)	O2'—C11'—C12'—O3'	−0.1 (5)
C10—C11—C12—O3	178.2 (5)	C10'—C11'—C12'—O3'	179.1 (5)
O2—C11—C12—C13	178.7 (5)	O2'—C11'—C12'—C13'	−179.2 (4)
C10—C11—C12—C13	0.0	C10'—C11'—C12'—C13'	0.0
C16—O4—C13—C12	−2.2 (7)	C16'—O4'—C13'—C14'	177.9 (7)
C16—O4—C13—C14	178.9 (6)	C16'—O4'—C13'—C12'	−2.3 (8)
O3—C12—C13—O4	2.6 (5)	O3'—C12'—C13'—O4'	1.0 (5)
C11—C12—C13—O4	−179.0 (4)	C11'—C12'—C13'—O4'	−179.8 (5)
O3—C12—C13—C14	−178.4 (4)	O3'—C12'—C13'—C14'	−179.2 (4)
C11—C12—C13—C14	0.0	C11'—C12'—C13'—C14'	0.0
O4—C13—C14—O5	−0.4 (4)	O4'—C13'—C14'—O5'	−0.9 (5)
C12—C13—C14—O5	−179.5 (4)	C12'—C13'—C14'—O5'	179.3 (4)
O4—C13—C14—C10	179.1 (4)	O4'—C13'—C14'—C10'	179.8 (4)
C12—C13—C14—C10	0.0	C12'—C13'—C14'—C10'	0.0
C9—C10—C14—O5	0.5 (5)	C9'—C10'—C14'—O5'	−0.5 (6)
C11—C10—C14—O5	179.5 (4)	C11'—C10'—C14'—O5'	−179.3 (4)
C9—C10—C14—C13	−178.9 (4)	C9'—C10'—C14'—C13'	178.8 (4)
C11—C10—C14—C13	0.0	C11'—C10'—C14'—C13'	0.0

### Hydrogen-bond geometry (Å, °)

**Table:**

*D*—H···*A*	*D*—H	H···*A*	*D*···*A*	*D*—H···*A*
O1—H1···O2	0.84	1.93	2.600 (5)	136
O1'—H1'···O2'	0.84	1.97	2.623 (4)	134
C2—H2···O2^i^	0.95	2.26	3.091 (8)	145
C16'—H16D···O1'^ii^	0.98	2.05	2.885 (11)	142

Symmetry codes: (i) *x*, *y*+1, *z*; (ii) *x*, *y*−1, *z*.
